# A Novel Role of *Eruca sativa* Mill. (Rocket) Extract: Antiplatelet (NF-κB Inhibition) and Antithrombotic Activities

**DOI:** 10.3390/nu6125839

**Published:** 2014-12-12

**Authors:** Eduardo Fuentes, Marcelo Alarcón, Manuel Fuentes, Gilda Carrasco, Iván Palomo

**Affiliations:** 1 Department of Clinical Biochemistry and Immunohematology, Faculty of Health Sciences, Interdisciplinary Excellence Research Program on Healthy Aging (PIEI-ES), Universidad de Talca, Talca 3460000, Chile; E-Mails: edfuentes@utalca.cl (E.F.); malarcon@utalca.cl (M.A.); mfuentes@alumnos.utalca.cl (M.F.); 2 Centro de Estudios en Alimentos Procesados (CEAP), CONICYT-Regional, Gore Maule 3460000, R09I2001, Chile; 3 Horticulture Department, Faculty of Agricultural Sciences, Universidad de Talca, Talca 3460000, Chile

**Keywords:** *Eruca sativa* Mill., rocket, antiplatelet, antithrombotic, platelet inflammatory mediators, functional food

## Abstract

Background: Epidemiological studies have shown the prevention of cardiovascular diseases through the regular consumption of vegetables. *Eruca sativa* Mill., commonly known as rocket, is a leafy vegetable that has anti-inflammatory activity. However, its antiplatelet and antithrombotic activities have not been described. Methods: *Eruca sativa* Mill. aqueous extract (0.1 to 1 mg/mL), was evaluated on human platelets: (i) P-selectin expression by flow cytometry; (ii) platelet aggregation induced by ADP, collagen and arachidonic acid; (iii) IL-1β, TGF-β1, CCL5 and thromboxane B2 release; and (iv) activation of NF-κB and PKA by western blot. Furthermore, (v) antithrombotic activity (200 mg/kg) and (vi) bleeding time in murine models were evaluated. Results: *Eruca sativa* Mill. aqueous extract (0.1 to 1 mg/mL) inhibited P-selectin expression and platelet aggregation induced by ADP. The release of platelet inflammatory mediators (IL-1β, TGF-β1, CCL5 and thromboxane B2) induced by ADP was inhibited by *Eruca sativa* Mill. aqueous extract. Furthermore, *Eruca sativa* Mill. aqueous extract inhibited NF-κB activation. Finally, in murine models, *Eruca sativa* Mill. aqueous extract showed significant antithrombotic activity and a slight effect on bleeding time. Conclusion: *Eruca sativa* Mill. presents antiplatelet and antithrombotic activity.

## 1. Introduction

Commonly found in the Mediterranean diet, the rocket species is from the family, *Brassicaceae*. The species includes *Eruca sativa* Mill., *Diplotaxis* species and *Bunias orientalis*, which are eaten at different ontogenic stages [1]. The positive and beneficial effects of the phytochemicals contained in rocket on human health have been reported by several clinical research studies. Moreover, the consumption of green leafy vegetables has been associated with a reduced risk of cardiovascular diseases (CVD) [2]. These beneficial effects have been attributed to the range of phytochemicals they contain, including vitamins A and C, flavonoids and glucosinolates, all of which are found in high levels in *Brassicaceae* crops [3,4].

*Eruca sativa* Mill. (commonly known as rocket) is a fast-growing and cool-season crop. The levels can be cut after 20 days and are sequentially harvested from re-growth [5]. Rocket is considered an excellent source of antioxidants, as it includes phenolic compounds, carotenoids, glucosinolates and degradation products, such as isothiocyanates [6]. Furthermore, *Eruca sativa* Mill. possesses anti-secretory, anti-inflammatory, cytoprotective and anti-ulcer activity against experimentally-induced gastric lesions. The anti-ulcer effect is possibly due to prostaglandin-mediated activity and/or through anti-secretory and antioxidant properties [7,8].

Platelet adhesion, activation and aggregation on the exposed subendothelial extracellular matrix are essential for hemostasis, but may also lead to thrombosis [9]. Platelet activation triggers the synthesis and release of several autocrine and paracrine mediators, including adenosine diphosphate (ADP), thrombin, epinephrine and thromboxane A2 (TXA2) [10,11]. These agonists activate multiple protein-mediated signaling pathways, inducing platelet shape change, degranulation and integrin activation [12]. Hence, platelet activation is the initial and central event leading to atherothrombosis after the rupture of atheromatous plaques [13].

Despite the important role of platelet activation in the development of acute thrombosis and CVD, no data are available regarding the effect of *Eruca sativa* Mill. on platelet activation and thrombus formation. The aim of this study was to investigate the antiplatelet and antithrombotic activities of aqueous extract from rocket.

## 2. Experimental Section

### 2.1. Reagents and Antibodies

The agonists ADP and arachidonic acid (AA), acetylsalicylic acid (ASA), rose bengal and prostaglandin E_1_ (PGE_1_) were obtained from Sigma-Aldrich (St. Louis, MO, USA), and collagen was obtained from Hormon-Chemie (Munich, Germany). Dimethyl sulfoxide (DMSO), HEPES, methanol and anti-phospho-NF-κB p65 were obtained from Sigma-Aldrich (St. Louis, Missouri/MO, USA). Anti-phospho-protein kinase A (PKA) antibody was obtained from Santa Cruz (Biotechnology, California, CA, USA). Anti γ-tubulin monoclonal antibody (4D11) was obtained from Thermo Scientific (Thermo Scientific, Pierce, Rockford, IL, USA). Antibodies (anti-CD62P-PE and anti-CD61-FITC) were obtained from BD Pharmingen (BD Biosciences, San Diego, CA, USA). DMSO 0.2% was employed as the vehicle for the preparation of working solutions of *Eruca sativa* Mill. extract.

### 2.2. Processing Material

*Eruca sativa* Mill. cv. Sauvage leaves were harvested from a crop obtained from a commercial hydroponic company in the Region of Maule, Chile, 25 days after sowing. The float Speedling system was used to grow this crop [14,15].

### 2.3. Preparation of Extract

Extract from *Eruca sativa* Mill. was obtained according to Fuentes *et al.* [16]. In brief, the samples were comminuted in a blender and mixed with water:methanol, 7:3 v/v, then sonicated and centrifuged for 10 min at 700× *g*. Then, the supernatant was lyophilized at −45 °C (freeze dried) and stored at −80 °C until use.

### 2.4. Preparation of Human Platelet Suspensions

After receiving written informed consent, venous blood samples were taken from six young healthy volunteers. The protocol was authorized by the ethics committee of the Universidad de Talca in accordance with the Declaration of Helsinki (approved by the 18th World Medical Assembly in Helsinki, Finland, 1964). The samples were placed in 3.2% citrate tubes (9:1 v/v) by phlebotomy with a vacuum tube system (Becton Dickinson Vacutainer Systems, Franklin Lakes, NJ, USA). Samples obtained from each volunteer were processed independently for each assay and centrifuged (DCS-16 Centrifugal Presvac RV) at 240× *g* for 10 min to obtain platelet-rich plasma (PRP). Subsequently, two-thirds of PRP were removed and centrifuged (10 min at 650× *g*). The pellet was then washed with HEPES-Tyrode’s buffer containing PGE_1_ (120 nmol/L). Washed platelets were prepared in HEPES-Tyrode’s buffer at a concentration of 200 × 10^9^ platelets/L (Bayer Advia 60 Hematology System, Tarrytown, NY, USA). After blood samples were taken, platelets were kept at 4 °C during all of the isolation steps.

### 2.5. Flow Cytometry Analysis for P-Selectin

P-selectin expression on platelet surface was analyzed by flow cytometry [17]. Briefly, 480 µL of washed platelets were pre-incubated with 20 µL of vehicle (DMSO 0.2%) or *Eruca sativa* Mill. extract (0.1 to 1 mg/mL) for 3 min, followed by 6 minutes of stimulation at 37 °C with ADP 8 μmol/L. To determine platelet P-selectin expression, 50 µL of the sample were mixed with saturated concentrations of anti-CD62P-PE and anti-CD61-FITC and incubated for 25 min in the dark. Samples were then acquired and analyzed in an Accuri C6 flow cytometer (BD, Biosciences, San Diego, CA, USA). Platelet populations were gated on cell size using forward scatter (FSC) *vs*. side scatter (SSC) and CD61 positivity to distinguish them from electronic noise. The light scatter and fluorescence channels were set at logarithmic gain, and 5000 events per sample were analyzed. Fluorescence intensities of differentially-stained populations were expressed as the mean channel value using the BD Accuri C6 Software (BD Biosciences, San Diego, CA, USA). All measurements were performed from six separate platelet donors.

### 2.6. Measurement of Platelet Aggregation

Platelet aggregation was monitored by light transmission according to Born and Cross [18], using a lumi-aggregometer (Chrono-Log, Havertown, PA, USA). Briefly, 480 μL of PRP in the reaction vessel were pre-incubated with 20 μL of vehicle (DMSO 0.2%) or *Eruca sativa* Mill. extract (0.1 to 1 mg/mL). After 3 min of incubation, 20 μL of agonist (ADP 8 µmol/L, collagen 1.5 μg/mL or AA 1 mmol/L) were added to initiate platelet aggregation, which was measured for 6 min. The platelet aggregation (maximal amplitude (%)) was determined by AGGRO/LINK software (Chrono-Log, Havertown, PA, USA). The inhibition of the maximal platelet aggregation was expressed as a percentage with respect to control (DMSO 0.2%). The concentration required to inhibit platelet aggregation by 50% (IC_50_) was calculated from the dose-response curves. All measurements were performed from six separate platelet donors.

### 2.7. Measurement of Thromboxane B2, CCL5, TGF-1β and IL-1β Levels

Thromboxane B2, CCL5, TGF-1β and IL-1β levels were determined using human Quantikine ELISA kits (R&D systems, Minneapolis, MN, USA). Briefly, washed platelets (200 × 10^9^ platelets/L) were pretreated with vehicle (DMSO 0.2%) or *Eruca sativa* Mill. extract (0.1 to 1 mg/mL) for 15 min at 37 °C and then stimulated by ADP (8 μmol/L) for 15 min at 37 °C. Finally, the supernatants were collected after centrifugation at 11,000× *g* for 10 min at 4 °C and stored at −70 °C until use. The thromboxane B2, CCL5, TGF-1β and IL-1β levels of the supernatants were measured using ELISA kits. All measurements were performed from six separate platelet donors.

### 2.8. Measurement of cAMP Levels in Human Platelets

The effect of *Eruca sativa* Mill. extract (0.1 to 1 mg/mL) on cAMP platelet levels was evaluated in 480 µL of washed platelets (200 × 10^9^ platelets/L) after a 5-min incubation period without stirring. The platelet reaction was stopped in ice-cold 15% trichloroacetic acid, and precipitated proteins were removed by centrifugation. Samples were stored at −70 °C until analysis. Before determination, samples were dissolved in 200 µL PBS at pH 6.2. The cAMP Parameter Assay Kit (R&D Systems, Minneapolis, MN, USA) was used. All measurements were performed from six separate platelet donors.

### 2.9. Western Blotting

Washed platelets (200 × 10^9^ platelets/L) were pre-incubated with vehicle (DMSO 0.2%) or *Eruca sativa* Mill. extract (0.1 to 1 mg/mL) for 3 min and activated with ADP (8 µmol/L) for 6 min. Then, platelets were lysed with 0.2 mL of lysis buffer in ice for 30 min and heated for 10 min at 95 °C. Equal quantities of total protein (30 μg) were subjected to SDS-PAGE under reducing conditions and transferred to a nitrocellulose membrane. The proteins were detected with anti-phospho-PKA, anti-phospho-NF-κB p65 and anti-γ-tubulin antibodies. All measurements were performed from six separate platelet donors.

### 2.10. Murine Model of Thrombosis 

This study was carried out under recommendations by the Guide for the Care and Use of Laboratory Animals of the National Institutes of Health. The protocol was approved by the Committee on the Ethics of Animal Experiments of the University of Talca. All efforts were made to minimize suffering. Thrombosis in mice was performed by photochemical injury using modified methods described by Przyklenk and Whittaker [19]. Briefly, C57BL/6 mice (12–16 weeks old) were anesthetized with a combination of tribromoethanol (270 mg/kg) and xylazine (13 mg/kg). Thrombosis was induced by an injection of 50 mg/kg rose bengal through the tail vein followed by illumination of the exposed mesenteric artery with a 1.5-mW green light laser (532 nm). Blood flow was monitored for 60 min, and stable occlusion was defined as a blood flow of 0 mL/min for 3 min. *Eruca sativa* Mill. extract (200 mg/kg, *n* = 6), vehicle (DMSO 0.2% group, *n* = 6) and ASA (200 mg/kg, *n* = 6) were administered intraperitoneally 30 min before the experiment. After laser exposure, the injury image generated was recorded with a charge-coupled device camera (Lumenera Corporation, Ottawa, ON, Canada). The image was analyzed with ImageJ software (version 1.26t, NIH, USA). The region of interest (vessel occlusion) was defined as the target artery, which included the portion of the target artery that was larger than the maximum injured area. Using software tools, thrombus size was measured in the region of interest. Rectal temperatures were similar and within the physiological range between all experimental animals throughout the experimental period.

### 2.11. Bleeding Assay

C57BL/6 mice were anesthetized with a combination of tribromoethanol (270 mg/kg) and xylazine (13 mg/kg) and placed prone on a warming pad from which the tail protruded. The same amounts of *Eruca sativa* Mill. extract (200 mg/kg, *n* = 6, intraperitoneally), ASA (200 mg/kg, *n* = 6, intraperitoneally) or vehicle (DMSO 0.2%, *n* = 6, intraperitoneally) were given as described in the thrombosis model. An incision was made on the ventral surface of the mice tails about 2 mm from the tip [20]. The bleeding time was measured in seconds (s) until bleeding stopped.

### 2.12. Statistical Analysis

Data were analyzed using SPSS version 17.0 (SPSS, Inc., Chicago, IL, USA) and expressed as the mean ± standard error of mean (SEM). Six or more independent experiments were performed for the different assays. Results were expressed as a percentage of inhibition or as a percentage of control (as 100%). The fifty-percent inhibitory concentration (IC_50_) of *Eruca sativa* Mill. extract was calculated from the dose-response curves. Differences between groups were analyzed by a one-way analysis of variance (ANOVA) using Tukey’s *post hoc* test. *p*-values <0.05 were considered significant.

## 3. Results

### 3.1. Effect of Eruca sativa *Mill.* Extract on Platelet Activation 

**Figure 1:**
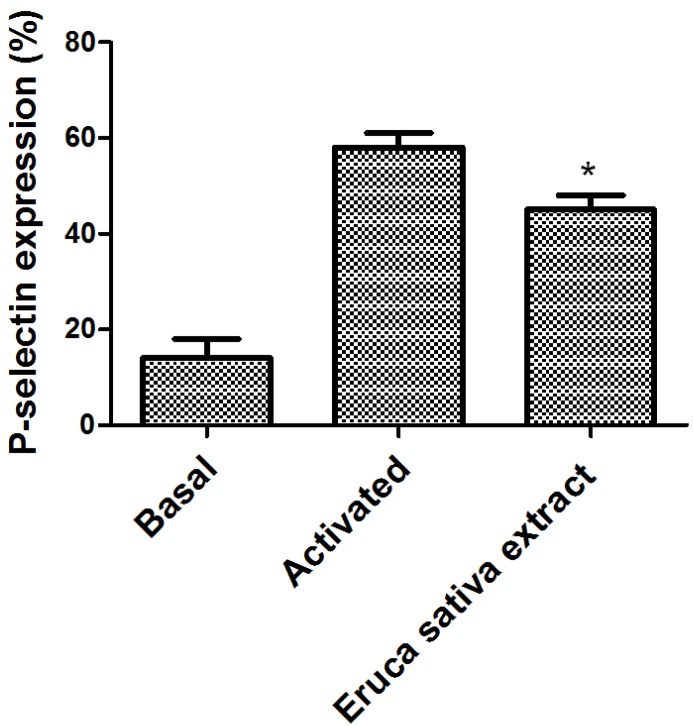
Effect of *Eruca sativa* Mill. extract on platelet activation. P-selectin expression was determined by flow cytometry. The basal bar represents the fluorescence in the non-stimulated sample. The graph depicts the mean ± SEM of *n* = 6 experiments. An asterisk (* *p* < 0.05) denotes a statistically significant difference when *Eruca sativa* Mill. extract was compared with the activated control analyzed by one-way ANOVA and Tukey’s *post hoc* test.

The effect of *Eruca sativa* Mill. extract on P-selectin expression in human platelets after stimulation by ADP in PRP was measured by flow cytometry (Figure 1). In the presence of *Eruca sativa* Mill. extract (1 mg/mL), the P-selectin expression was inhibited from 58 ± 3 to 45 ± 3% (*p* < 0.05).

### 3.2. Effect of Eruca sativa *Mill.* Extract on Platelet Aggregation

**Figure 2:**
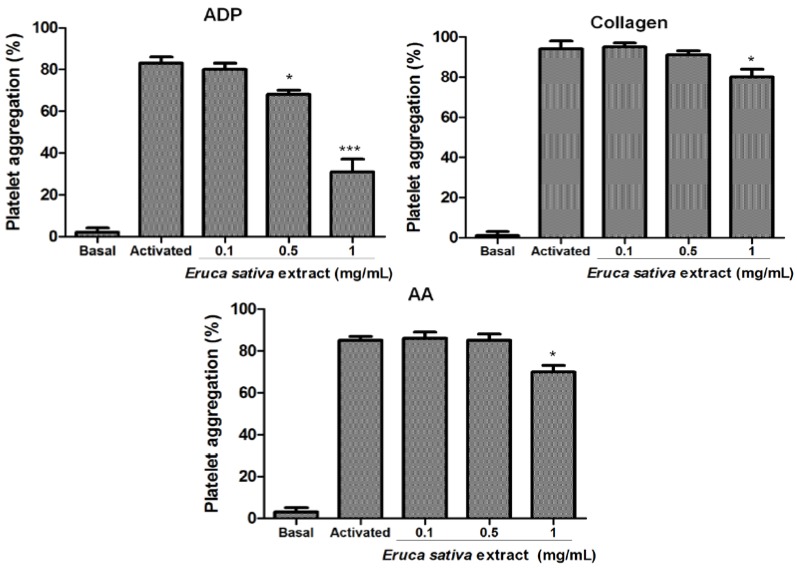
Effects of *Eruca sativa* Mill. extract on ADP (8 μmol/L), collagen (1.5 µg/mL) and AA (1 mmol/L) induced platelet aggregation. Results were expressed as platelet aggregation (%) (mean ± SEM, *n* = 6). Asterisks (* *p* < 0.05 and *** *p* < 0.001) denote a statistically significant difference when compared with the activated control analyzed by one-way ANOVA and Tukey’s *post hoc* test. AA = arachidonic acid.

The effects of *Eruca sativa* Mill. extract on platelet aggregation induced by ADP, collagen and AA are shown in Figure 2. *Eruca sativa* Mill. extract inhibited ADP-induced platelet aggregation with a 50% inhibitory concentration (IC_50_) of 0.71 mg/mL. In addition, *Eruca sativa* Mill. extract only showed a mild inhibitory effect (17 ± 4 and 16% ± 3%, *p* < 0.05) over collagen and AA-induced platelet aggregation at a concentration of 1 mg/mL.

### 3.3. Effects of Eruca sativa *Mill.* Extract on Thromboxane B2, CCL5, TGF-1β and IL-1β Levels

**Figure 3:**
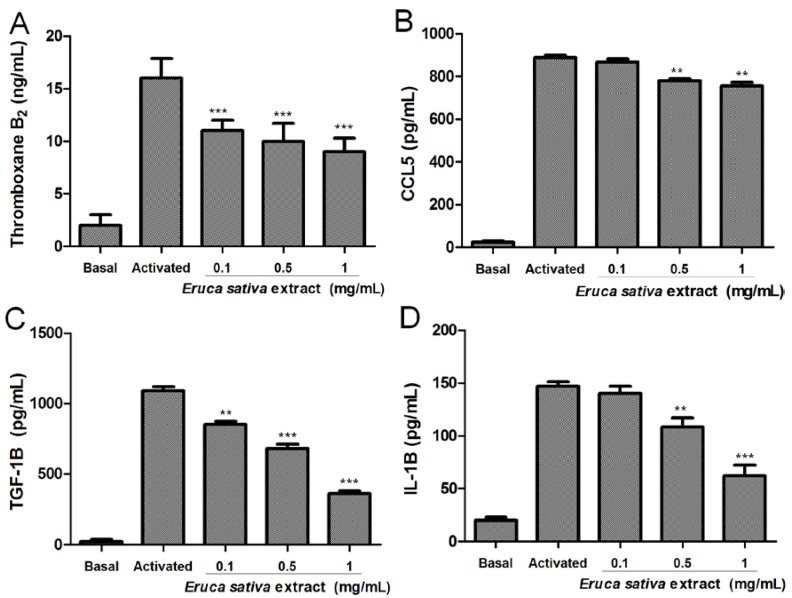
Effects of *Eruca sativa* Mill. extract on the release of thromboxane B_2_ (**A**), CCL5 (**B**), TGF-β1 (**C**) and IL-1β (**D**) induced by ADP (8 μmol/L) from human platelets. The graph depicts the mean ± SEM of *n* = 6 experiments. ** *p* < 0.01 and *** *p* < 0.001 indicates differences when compared with activated control, analyzed by one-way ANOVA and Tukey’s *post hoc* test.

As shown in Figure 3, resting platelets produced relatively low thromboxane B2, CCL5, TGF-1β and IL-1β levels compared to ADP-activated platelets. *Eruca sativa* Mill. extract (0.1 to 1 mg/mL) concentration-dependently inhibited thromboxane B2, CCL5, TGF-1β and IL-1β levels in platelets stimulated by ADP (8 μmol/L).

In washed platelets, ADP-induced thromboxane B2 release in the presence of *Eruca sativa* Mill. extract at 0.1, 0.5 and 1 mg/mL was inhibited from 16 ± 1.9 ng/mL in the control group to 11 ± 1, 10 ± 1.7 and 9 ± 1.3 pg/mL (*p* < 0.001), respectively (Figure 3A). In addition, we examined the effect of *Eruca sativa* Mill. extract on platelet CCL5 release. As observed in Figure 3B, *Eruca sativa* Mill. extract significantly reduced ADP-induced platelet CCL5 release from 888 ± 11 pg/mL in the control group to 780 ± 9 and 755 ± 17 pg/mL (*p* < 0.01), at concentrations of 0.5 and 1 mg/L, respectively. Furthermore, in this study, *Eruca sativa* Mill. extract at 0.1, 0.5 and 1 mg/mL inhibited the effect of ADP-induced TGF-1β release by 22 ± 1, 38 ± 2 and 67% ± 1% (*p* < 0.001), respectively (Figure 3C). Meanwhile, ADP-induced IL-1β release was inhibited by 26 ± 3 and 57% ± 3% (*p* < 0.001) in the presence of *Eruca sativa* Mill. extract at 0.5 and 1 mg/mL, respectively (Figure 3D).

### 3.4. Eruca sativa *Mill.* Extract and Intraplatelet Levels of cAMP

We investigated whether platelet inhibition by *Eruca sativa* Mill. extract was mediated by changes of intraplatelet cAMP levels. *Eruca sativa* Mill. extract (0.1 to 1 mg/mL) did not show any effect on intraplatelet levels of cAMP. Levels of cAMP in resting platelets were marked lower than those observed in PGE1 (0.02 mmol/L)-treated platelets (*p* < 0.001).

### 3.5. Effects of Eruca sativa *Mill.* Extract on PKA and NF-κB

**Figure 4:**
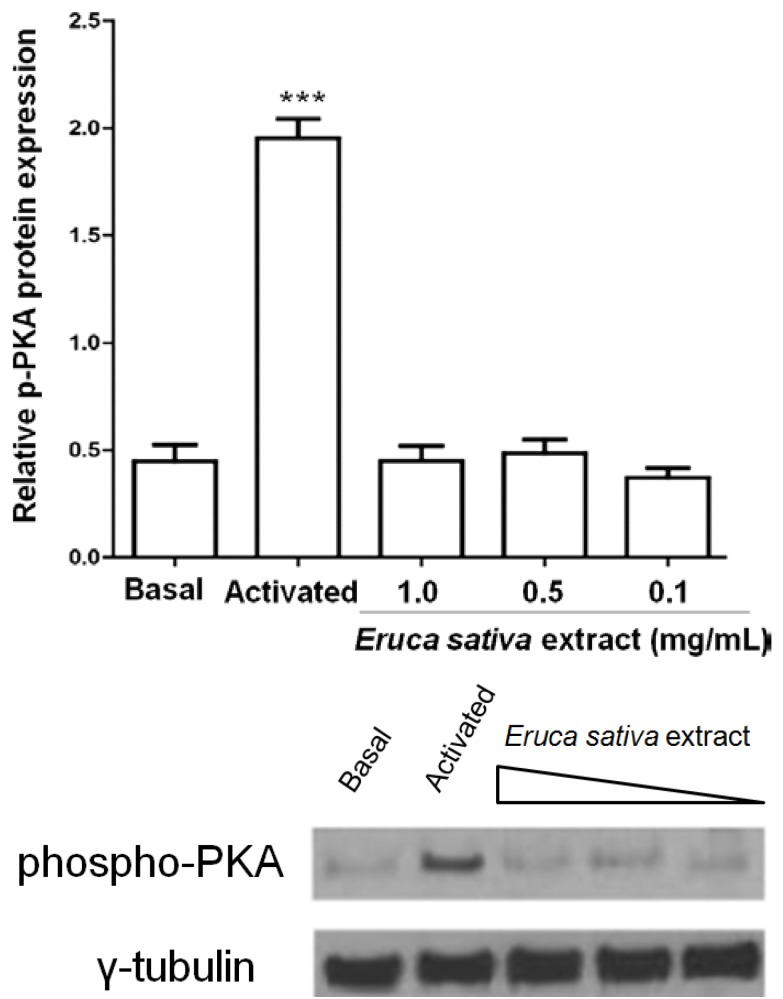
Effect of *Eruca sativa* Mill. extract on phospho-PKA in activated platelets by ADP (8 μmol/L). The activated group corresponds to prostaglandin E_1_ (PGE_1_) plus ADP. Data are presented as the mean ± SEM of *n* = 6 experiments. *** *p* < 0.001 indicates the difference between activated and basal groups as analyzed by one-way ANOVA and Tukey’s *post hoc* test.

PKA activation by cAMP phosphorylates multiple target proteins in numerous platelet inhibitory pathways. Here, the treatment of washed platelets with *Eruca sativa* Mill. extract (0.1 to 1 mg/mL) did not increase the phosphorylation of PKA (Figure 4).

**Figure 5:**
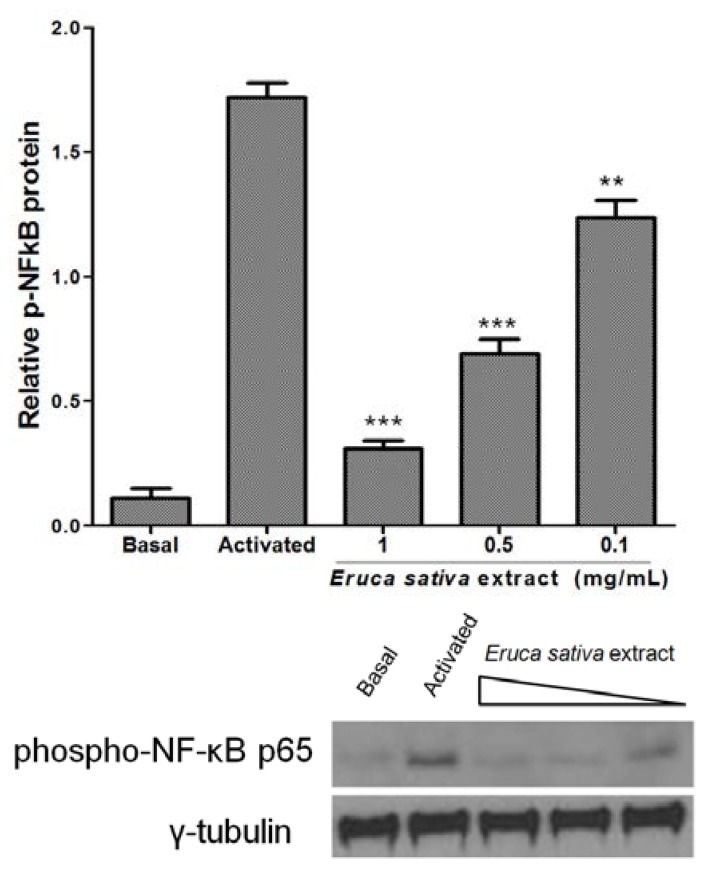
Effect of *Eruca sativa* Mill. extract on phospho-NF-κB p65 in activated platelets by ADP (8 μmol/L). Washed platelets were collected, and subcellular extracts were analyzed for phospho-NF-κB p65, as described in the Experimental Section. Data are presented as the mean ± SEM of *n* = 6 experiments. ** *p* < 0.01 and *** *p* < 0.001 indicates differences between activated and *Eruca sativa* Mill. extract groups as analyzed by one-way ANOVA and Tukey’s *post hoc* test.

Platelets express three members of the NF-κB pathway: IKK, IκB and phospho-NF-κB p65 [21]. The present study demonstrated that p65 phosphorylation was markedly increased in ADP-induced activation of washed platelets, and *Eruca sativa* Mill. extract (0.1 to 1 mg/mL) concentration-dependently attenuated phospho-NF-κB p65 in platelets stimulated by ADP (8 μmol/L) (Figure 5).

### 3.6. Effect of Eruca sativa *Mill.* Extract on Arterial Thrombus Formation and Bleeding Time

**Figure 6:**
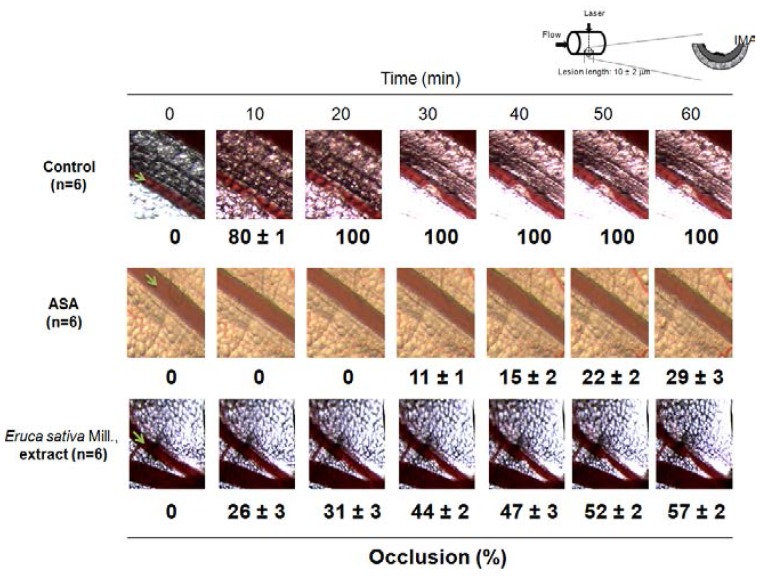
*Eruca sativa* Mill. extract inhibited arterial thrombosis formation. Representative images of thrombus formation after laser irradiation in the vehicle (DMSO 0.2%) control group (*n* = 6), ASA (acetylsalicylic acid; 200 mg/kg; *n* = 6) and *Eruca sativa* extract (200 mg/kg, *n* = 6) to 60 min. Each photograph represents the percentage of occlusions caused by thrombus formation. I, intima; M, media; A, adventitia.

As shown in Figure 6, the mesenteric artery of untreated mice (control) was completely occluded by a stable bulky thrombus 30 min after laser injury. In contrast, one intraperitoneal bolus injection of *Eruca sativa* Mill. extract (200 mg/kg) delayed vessel occlusion to 60 min and reduced the maximum occlusion (occlusion for 100%) to 57% ± 2% (*p* < 0.01).

We measured *Eruca sativa* Mill. extract-induced C57BL/6 mouse blood loss after tail snip at the same concentration that was used for arterial thrombus formation *in vivo* (200 mg/kg, a single bolus intraperitoneally injection). In this study, the same antithrombotic concentration used of *Eruca sativa* Mill. extract did not cause significant bleeding measured by tail snip. Thus, the bleeding time by *Eruca sativa* Mill. extract of 187 ± 19 s (*n* = 6) was not statistically significantly higher than the control (171 ± 26 s, *n* = 6) (*p* > 0.05).

Concurrently, ASA (200 mg/kg) reduced the maximum occlusion (occlusion for 100%) to 29% ± 3% (*p* < 0.01). However, at the same antithrombotic concentration, the bleeding time by ASA of 290 ± 23 s (*n* = 6) was statistically significantly higher than the control (171 ± 26 s, *n* = 6) (*p* < 0.05).

## 4. Discussion

In this study, we demonstrated for the first time that *Eruca sativa* Mill. extract inhibits platelet aggregation and activation, reduces platelet release of atherosclerotic-related inflammatory mediators (thromboxane B2, CCL5, TGF-1β and IL-1β levels) and decreases *in vivo* thrombus formation. In addition, evidence is provided that *Eruca sativa* Mill. extract antiplatelet activities are associated with NF-κB inhibition.

Although antiplatelet drugs have a cardiovascular protective function, many also have side effects (including headaches, gastrointestinal symptoms, skin rash and bleeding) [22]. Therefore, there is an urgent need to identify more effective and safe antiplatelet and antithrombotic agents. In this way, herbs, medicinal plants, spices and vegetables are a potential source to help combat various diseases, including CVD. In recent years, *Eruca sativa* Mill. rocket leaves, a member of the *Brassicaceae* family, have been eaten (at different ontogenic stages) all over the world. The leaves are eaten raw or cooked, and *Eruca sativa* Mill. flowers are also consumed [23,24]. The seeds, roots, leaves and flowers of *Eruca sativa* Mill. contain different flavonoid profiles [1]. Kaempferol derivatives represent the major group of phenolics present in *Eruca sativa* Mill. leaves (77%–88% of total phenolics), followed by quercetin and isorhamnetin-3,4-diglucoside, representing 9% and 16.3% of the total phenolics, respectively [25,26]. The antiplatelet activity of *Eruca sativa* Mill., could be by the presence of kaempferol, quercetin and isorhamnetin [27,28,29].

In this study, *Eruca sativa* Mill. extract displayed *in vitro* and *in vivo* antiplatelet activities. Thus, *Eruca sativa* Mill. extract significantly inhibits platelet activation (less P-selectin expression) and platelet aggregation induced by ADP and showed only a low inhibition over collagen and AA.

Platelet inflammatory mediators (thromboxane B2, CCL5, TGF-1β and IL-1β) contribute to atherosclerotic lesion development and arterial thrombogenesis [30]. These results show that *Eruca sativa* Mill. extract inhibited human platelet thromboxane B2, TGF-1β and IL-1β levels induced by ADP. Furthermore, to a lesser extent, *Eruca sativa* Mill. extract inhibited human platelet CCL5 levels induced by ADP.

Although platelets are anucleated cells, they express several transcription factors that exert non-genomic functions (*i.e.*, NF-κB) [31]. Platelet activation triggers IκBα phosphorylation and degradation and NF-κB phosphorylation [21,31]. NF-κB phosphorylation allows platelet adhesion to fibrinogen, glycoprotein IIb/IIIa activation, P-selectin expression and TxA2 formation, which was also demonstrated [31]. In the study, *Eruca sativa* Mill. extract markedly inhibited NF-κB activation in ADP-stimulated platelets. 

Using a murine model of real-time thrombus formation [32], we demonstrated, for the first time, that *Eruca sativa* Mill. extract prevented thrombus growth *in vivo*. Moreover, antiplatelet drugs that are currently available inevitably increase bleeding risk at antithrombotic doses [33]. This study showed that *Eruca sativa* Mill. extract possesses antithrombotic efficacy without significant bleeding.

## 5. Conclusions

Rocket extract shows antiplatelet activity (inhibition of platelet activation, aggregation and release of inflammatory mediators), and the mechanism of action could be by NF-κB inhibition. However, further studies are needed to expand *Eruca sativa* Mill. extract properties in the setting of the NF-κB pathway.
